# Sensitive PCR method for the detection and real-time quantification of human cells in xenotransplantation systems

**DOI:** 10.1038/sj.bjc.6600573

**Published:** 2002-11-12

**Authors:** M Becker, A Nitsche, C Neumann, J Aumann, I Junghahn, I Fichtner

**Affiliations:** Max-Delbrück-Center for Molecular Medicine, Robert-Rössle-Strasse 10, 13092 Berlin, Germany; Experimental Pharmacology & Oncology GmbH, Robert-Rössle-Strasse 10, 13125 Berlin, Germany; Clinics for Internal Medicine, Department Haematology and Oncology, Charité - Campus Virchow, Humboldt University, Augustenburger Platz 1, 13353 Berlin, Germany

**Keywords:** TaqMan PCR, xenografts, metastasis, leukaemia, haematological cells

## Abstract

The sensitive detection of human cells in immunodeficient rodents is a prerequisite for the monitoring of micrometastasis of solid tumours, dissemination of leukaemic cells, or engraftment of haematological cells. We developed a universally applicable polymerase chain reaction method for the detection of a human-specific 850-bp fragment of the α-satellite DNA on human chromosome 17. The method allows the detection of one human cell in 10^6^ murine cells and could be established as both, a conventional DNA polymerase chain reaction-assay for routine screening, and a quantitative real-time polymerase chain reaction-assay using TaqMan-methodology. It was applied to the following xenotransplantation systems in SCID and NOD/SCID mice: (1) In a limiting dilution assay, cells of the MDA-MB 435 breast carcinoma were injected into the mammary fat pad of NOD/SCID mice. It could be shown that 10 cells mouse^−1^ were sufficient to induce a positive polymerase chain reaction signal in liver and lung tissue 30 days after transplantation as an indicator for micrometastasis. At this time a palpable tumour was not yet detectable in the mammary fat pad region. (2) Cells of a newly established human acute lymphatic leukaemia were administered intraperitoneally to SCID mice. These cells apparently disseminated and were detectable as early as day 50 in the peripheral blood of living mice, while the leukaemia manifestation was delayed by day 140. (3) In a transplantation experiment using mature human lymphocytes we wanted to standardise conditions for a successful survival of these cells in NOD/SCID mice. It was established that at least 5×10^7^ cells given intravenously were necessary and that the mice had to be conditioned by 2 Gy body irradiation to get positive polymerase chain reaction bands in several organs. (4) Engraftment studies with blood stem cells originating from cytapheresis samples of tumour patients or from cord blood were undertaken in NOD/SCID mice in order to define conditions of successful engraftment and to use this model for further optimisation strategies. The polymerase chain reaction method presented allowed a reliable prediction of positive engraftment and agreed well with the results of immunohistochemical or FACS analysis. All together, the polymerase chain reaction method developed allows a sensitive and reliable detection of low numbers of human cells in immunodeficient hosts. In combination with real-time (TaqMan) technique it allows an exact quantification of human cells. As this method can be performed with accessible material of living animals, follow up studies for the monitoring of therapeutic interventions are possible in which the survival time of mice as evaluation criteria can be omitted.

*British Journal of Cancer* (2002) **87**, 1328–1335. doi:10.1038/sj.bjc.6600573 www.bjcancer.com

© 2002 Cancer Research UK

## 

The detection of human cells in a murine tissue is a frequently arising problem after xenotransplantation of normal or malignant cells. The formation of micrometastases of solid tumours, the dissemination of leukaemia cells or the engraftment potential of haematopoietic cells are examples for which a sensitive proof of the human-specific origin of cells is necessary to address biological, diagnostic or therapeutic questions.

In literature, for that purpose tissue-(cytokeratins) or tumour associated markers like mucins (Finn *et al*, 1995) or adhesion molecules (Giavazzi *et al*, 1994) are described. Also human-specific surface markers like HLA-DR (Goan *et al*, 1999) or human endogenous retroviral sequences (Ailles *et al*, 1999) are used. Others, artificially transfected cells with marker genes like lac Z (Hayashi *et al*, 1996; Krüger *et al*, 1999) or green fluorescent protein (Yang *et al*, 2000) for a detection of the specific gene product in the *in vivo* xenotransplantation system.

These methods are at least partially hampered by the fact that tumour markers are only (over-) expressed in a certain proportion of known malignancies. Immunohistochemical methods for the detection of surface markers are relatively insensitive and difficult to standardise. Furthermore, the use of artificial markers is frequently restrained by the low transfection efficacy of the cells. Therefore, we decided to develop a sensitive PCR assay which is universally applicable either for the detection of human cells in murine blood and organs or for a quantification of such cells via real-time (TaqMan) PCR. We targeted for that purpose a highly repetitive α-satellite DNA sequence of the centromer region of human chromosome 17 with primers which were specifically modified by us according to Warburton *et al* (1991). The sensitivity and specificity of that method was investigated and used for: (i) the detection of micrometastases of a human breast carcinoma xenograft; (ii) the estimation of dissemination of a human acute lymphatic leukaemia (ALL) model; (iii) the proof of distribution of mature lymphocytes and (iv) the evaluation of the engraftment potential of human blood stem cells.

## MATERIALS AND METHODS

### Extraction of DNA

Genomic DNA of mouse blood and organs was extracted using the QiaAmp blood and tissue kit respectively (Qiagen, Hilden, Germany). Briefly, the DNA of lysed cells was adsorbed onto a silica matrix, washed and eluted with QIAamp elution buffer by centrifugation.

### Conventional DNA PCR

The presence of human-specific DNA within the blood and organs of transplanted mice was confirmed by polymerase chain reaction (PCR) amplifying an 850-bp fragment of the α-satellite region of the human chromosome 17 using primers corresponding to the primer pair 17a1/17a2 as described by Warburton *et al* (1991). The primers were elongated to 25 nucleotides each for use at higher annealing temperatures. The sequences are shown in Table 1

**Table 1:**
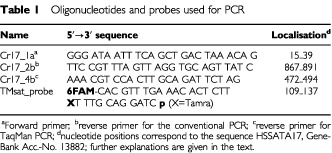
Oligonucleotides and probes used for PCR

. For PCR, the AmpliTaq-Gold polymerase and related reagents from Perkin Elmer (Applied Biosystems GmbH, Weiterstadt, Germany) were used. The PCR reaction mixture contained 200 μM each of the respective nucleotides, 250 nM of each primer, 2 mM MgCl_2,_ and 250 ng of genomic DNA template. Following an initial DNA denaturation and Taq activation at 94°C for 10 min, 35 1-min cycles of denaturation at 94°C and annealing/extension at 60°C were performed followed by a final elongation step at 72°C for 10 min. Amplified DNA fragments were electrophoresed through 1.75% agarose gels and subsequently visualised through ultraviolet light after staining with ethidium bromide. Genomic DNA samples from both a human breast carcinoma line (MaCa 3366) as a positive control and NOD/SCID mouse liver tissue as a negative control were processed in parallel. Routinely used PCR was evaluated by scoring band intensities expressed by one to three plus. One plus corresponded to very weak bands and three plus corresponded to very intense bands comparable to that of the positive control with 100% human cells.

### Quantitative real-time (TaqMan) PCR

In parallel, we developed a TaqMan-chemistry based real-time PCR using an optimised primer/probe-combination, which effectively amplifies a shorter (467 bp) fragment of the same α-satellite-DNA-region (Table 1). The exonuclease probe was 5′-labelled with the reporter fluorescent dye FAM (6-carboxy-fluoresceine) and carried the non-fluorescent quencher dye TAMRA. Probe extension during PCR was blocked by a 3′-phosphate.

The standardised PCR was performed in a Perkin Elmer 7700 Sequence Detection System (TaqMan) in 96-well microtiter plates with a final volume of 50 μl using similar conditions as for the conventional PCR described above. Additionally, the PCR-Mix contained 10 nM of the TaqMan-Probe. A total of 45 cycles was performed without a final elongation step. Each sample was tested twice in parallel. Serial dilution steps of human mammary carcinoma cells (MCF-7) in mouse ascitis cells (P388) served to construct the TaqMan-calibration curves. These control cells were obtained from *in vivo*-tumour lines established and permanently passaged in our laboratory.

### Experimental animals

For the *in vivo* experiments immunodeficient SCID or NOD/SCID mice (6–8 weeks of age) were used. While SCID mice lack mature B- and T-lymphocytes due to a recombinase gene deficiency (Bosma *et al*, 1983) NOD/SCID mice are additionally characterised by a deficit in NK-cells and functioning macrophages (Shultz *et al*, 1995). Balb/c-SCID mice were purchased from Bundesgesundheitsamt (Berlin, Germany) NOD/SCID mice from Jackson Laboratories (Bar Harbour, USA).

All mice were held under sterile and standardised conditions (20°C room temperature, 50% relative humidity, 12 h-light-dark rhythm) in laminar flow shelves with autoclaved food and bedding (Sniff, Soest, Germany) and acidified tap water (pH 4) *ad libitum*. All mice were tested for leakiness and only mice with murine IgG levels below 100 ng ml^−1^ serum were used for the experiments. All experiments were performed according to the German Animal Protection Law and with permission from the responsible local authorities.

### Detection of micrometastases

Cells of the human breast carcinoma MDA-MB 435 were taken from cell culture flasks and admixed with matrigel (Basement Membrane Matrix, Becton Dickinson, Bedford, USA) shortly before injection into the mammary fat pad (mfp) of female NOD/SCID mice. Six animals each received 10^6^ to 1 cell in a log 10 dilution assay. Once per week tumour size was measured with a calliper-like instrument and tumour volumes (v=(width^2^· length)/2) were calculated. Further, blood was taken from the tail vein once per week.

After 4 weeks mice were sacrificed, liver, lung and tumour from the mfp area were taken and stored at −80°C for PCR analysis.

### Leukaemic dissemination

Female Balb/c-SCID mice (five per group) received 10^2^, 10^4^ or 10^6^ cells of a human paediatric ALL (ALL-SCID 2, Fichtner *et al*, 1999) intraperitoneally (i.p.). Every 2–3 weeks blood was taken from the tail vein and stored for PCR analysis. Mice were checked at least twice per week for leukaemia development. Body weight was measured once per week and related to the initial value (body weight change, BWC) in per cent. The mice were sacrificed at moribund stage and gross examined.

### Distribution of lymphocytes

Three female NOD/SCID mice per group received 5×10^7^, 1×10^7^ or 0.5×10^7^ lymphocytes from a buffy coat after Ficoll gradient separation (Lymphocyte separation medium, Life Technologies, Eggenstein, Germany). The cell mixture contained 5% CD4+, 5% CD8+, 7% CD56+, 10% HLA-DR+ cells as determined by FACS analysis.

The lymphocytes were admixed with 10 U ml^−1^ heparin in order to prevent aggregation and injected intravenously (i.v.) into the tail vein. Half of the mice received a total body irradiation of 2 Gy (^137^Cs source) 24 h before lymphocyte administration in order to disrupt remaining circulatory macrophages in the circulation. After 4 weeks mice were sacrificed and different organs were analysed for human-specific DNA. In some mice a graft *vs* host disease (GVHD) was diagnosed by the appearance of ruffled fur, an icteric view of the skin and signs of inflammation in the head region. Livers of those mice were additionally examined by a pathologist for GVHD symptoms.

### Engraftment of blood stem cells

Mononuclear cells (MNC) originating from cord blood or from peripheral blood after stem cell mobilisation were injected i.v. into pre-irradiated (2 Gy) female NOD/SCID mice (2–5 per group). CD34 positive progenitors were obtained by magnetic separation as described elsewhere (Goan *et al*, 1996). The mice additionally received a co-transplant consisting of a rat fibroblast cell line stably transfected with the human interleukin-3 gene in order to supplement a species-specific haematopoietic growth factor (Goan *et al*, 1996). After at least 5 weeks the murine blood was analysed for human DNA. In the case of a positive PCR-signal mice were sacrificed 2–3 weeks later. Human haematopoietic cells (CD45+, HLA-I+) were analysed by flow cytometry (FACS) or immunohistochemistry (IH); for details see Goan *et al* (1996).

## RESULTS

### Sensitivity and specificity of PCR method

Figure 1

**Figure 1:**
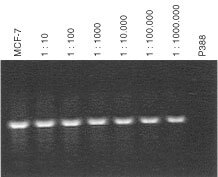
Human specific DNA-PCR of an α-satellite sequence of chromosome 17 used as a screening assay for human cells in the mouse. Sensitivity of the PCR method proved by logarithmic dilution steps of human MCF-7 cells in mouse P388 ascitis cells.

shows that at least a rough visual semi-quantitative evaluation of dilution mixtures of human in murine cells is possible by conventional DNA-PCR. While the murine P388 lymphatic leukaemia revealed no positive signal, as few as one human MCF-7 cell (breast carcinoma line) in 10^6^ of mouse cells was detectable. However, as our dilution experiments showed, the PCR method with 35 cycles seemed to reach a plateau phase. The differences between band intensities of logarithmic dilution steps were too small to perform a reliable densitometric analysis (data not shown). In contrast, the use of lower cycle numbers allowed a more reliable evaluation, but caused a disappearance of weak bands. Thus, for a routine screening we decided to perform a visual subjective estimation of band intensities characterised by one to three plus.

### TaqMan assay for real-time quantification of human DNA

**Figure 2:**
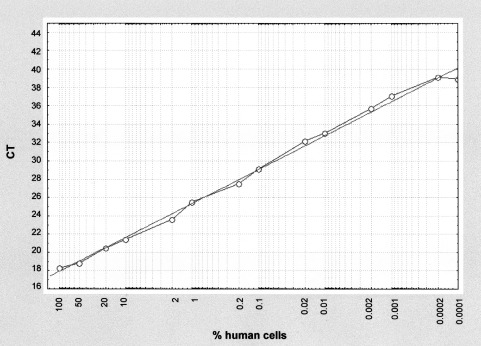
TaqMan created calibration curve using a dilution series of human MCF-7 cells in mouse P388 ascitis cells as processed with each TaqMan PCR. C_T_ describes the threshold cycle number at which fluorescence exceeds 10-fold the standard deviation of the fluorescence detected during PCR cycles 3–15. This calibration curve serves to calculate the amount of human DNA reflecting the number of human cells.

Since certain xenotransplantation experiments require an exact quantification of human cells in mouse organs, we developed in parallel a TaqMan-chemistry based real-time PCR approach amplifying a smaller fragment of the same genome localisation and using similar reaction and cycle conditions. Serial dilutions of human MCF-7 cells in mouse P388 cells tested with each PCR reaction served to construct the TaqMan-calibration curves. We always obtained a linear correlation between DNA-amount, respectively cell numbers, and the C_T_ values over the whole detection range from 50 to 0.001% human cells (with some slight deviations in the highest 100% and the lowest 0.0001% dilution step). Calibration results are presented in Figure 2

. Figure 3

**Figure 3:**
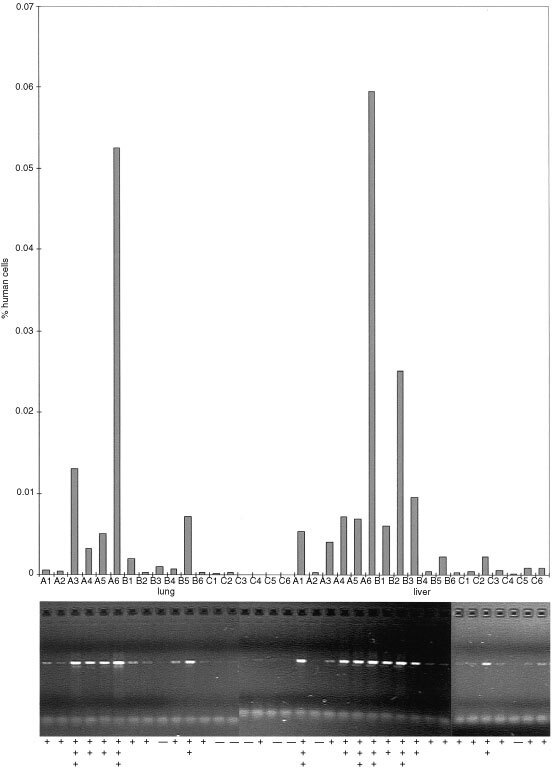
Example for the detection of micrometastases in mouse liver and lung in the MDA-MB 435 xenotransplantation experiment performed by both the conventional DNA PCR and the quantitative real-time PCR. The inoculation of 100 (**A**), 10 (**B**) and 1 (**C**) human tumour cells per mouse correlated with a decrease of the PCR signal in the organs (same as experiment documented in Table 2).

shows the TaqMan results of mouse liver and lung of three animal groups of the micrometastasis experiment with the human breast carcinoma xenograft MDA-MB 435 as described below (Table 2

**Table 2:**
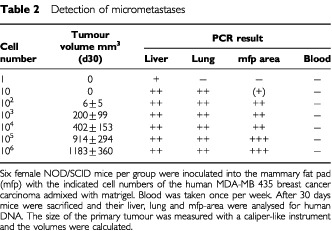
Detection of micrometastases

). The columns are related to the agarose gel bands of amplification products and the resulting visual estimation of band intensities of corresponding samples as obtained by conventional PCR.

### Detection of micrometastases

The human breast carcinoma line MDA-MB 435 is highly metastasising after orthotopic inoculation into the mfp of immunodeficient mice (Price *et al*, 1990). Lung and lymph node metastases are usually macroscopically detectable after a relatively long growth period when the primary tumour reaches a volume of more than 1.5 cm^3^.

In our assay system (Table 2 and Figure 3) only 10 cells per mouse were sufficient to detect human DNA in lung and liver of mice, while at that point of time (30 days after inoculation) no palpable tumour in the mfp-region was detectable. Higher cell numbers led to an increase in primary tumour size and a corresponding magnification of the PCR signal. Surprisingly, at no time point human DNA as indicator for circulating tumour cells could be found in the murine blood. This suggests either a predominant lymphogenic pathway of metastasis or indicates that cell numbers in circulation were below the detection limit of the assay. The PCR method presented here allows a very sensitive detection and quantification of micrometastases. It can be taken as surrogate marker for the monitoring of response of therapeutic interventions.

### Leukaemic dissemination

Table 3

**Table 3:**
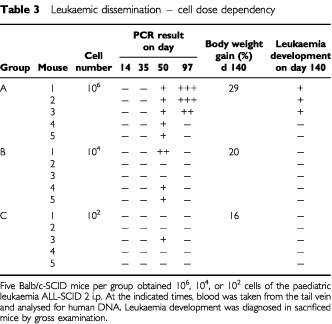
Leukaemic dissemination – cell dose dependency

shows the results of blood analysis of SCID mice at several time points after inoculation of cells of a paediatric leukaemia, ALL-SCID 2. While at day 50 transiently human DNA was detected in five out of five mice receiving 10^6^ cells, three out of five mice with 10^4^ and only one out of five mice with 10^2^ cells, at later time points (day 97) a positive PCR signal was only obtained in three out of five mice at the highest cell dose. At a delayed time, after 140 days, when the animals were sacrificed due to a moribund stage only in those mice with detection of human DNA at an advanced stage the leukaemia became manifest. The leukaemia development in the peritoneum was accompanied by a concomitant increase in body weight. It can be concluded that the determination of human DNA by the PCR method presented here, allows an early evaluation of leukaemic dissemination before manifestation of the disease. This enables the initiation of therapeutic interventions in the xenograft system at a stage being comparable with minimal residual disease in patients.

### Distribution of lymphocytes

The distribution pattern and survival rate of mature lymphocytes after i.v. injection into NOD/SCID mice can be taken from Table 4

**Table 4:**
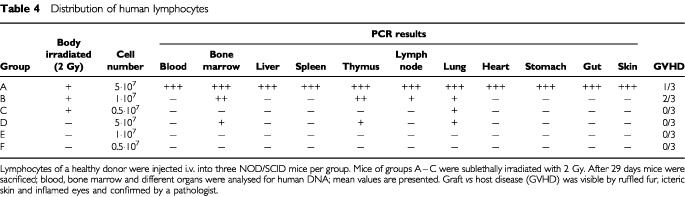
Distribution of human lymphocytes

. At least 10^7^ cells had to be inoculated to obtain a positive PCR signal in bone marrow, thymus, lymph node and lung, while the five-fold cell number apparently led to a broad distribution of lymphocytes to all tissues analysed. There was a clear difference between irradiated and non-irradiated mice indicating that a sublethal pre-irradiation leading to a long-standing depletion of macrophages (data not shown) is a precondition for a survival of i.v. inoculated mature lymphocytes.

The positive take rate of 5×10^7^ or 10^7^ lymphocytes was accompanied by the appearance of GVHD symptoms in one out of three or two out of three mice, respectively.

Our results confirm the limited survival potential of mature lymphocytes in the conditioned murine host. Oppositely, the PCR method enables the evaluation of lymphocyte distribution and survival in experiments investigating immunotherapeutic approaches against cancer.

### Engraftment of blood stem cells

The administration of cord blood mononuclear or CD34 positive (>90% of cell mixture) cells to NOD/SCID mice led in two out of three independently performed experiments (Table 5

**Table 5:**
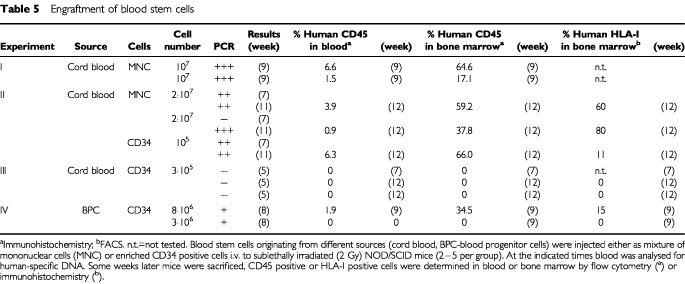
Engraftment of blood stem cells

**Figure 4:**
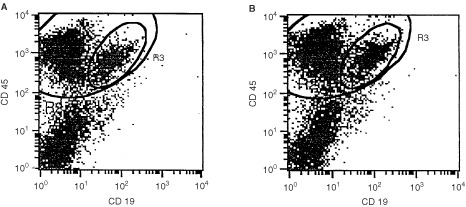
FACS analysis of bone marrow of mice 12 weeks after transplantation of cord blood MNC (**A**) or sorted CD 34 cells (**B**) from experiment II, Table 4. FL-1=CD 19^−^ FITC, FL-2=CD 45^−^ PE.

I and II) to positive PCR signals in the blood of mice at 7–11 weeks after transplantation. When blood and bone marrow of those mice were analysed for haematopoietic cells (CD45, HLA-I) by FACS and IH a high percentage of human cells could be found, especially in the bone marrow (Figures 4

and 5

**Figure 5:**
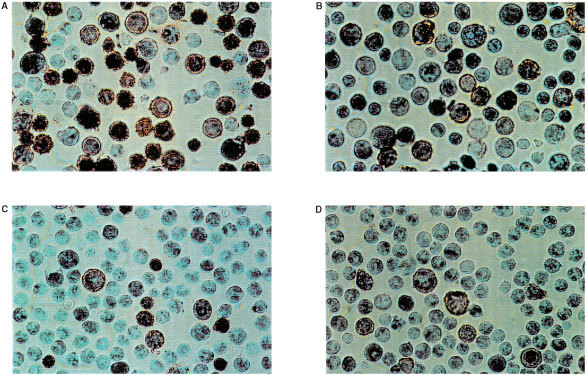
Immunohistochemistry of bone marrow of mice 12 weeks after transplantation of cord blood MNC (**A**, **B**) or CD 34 cells (**C**, **D**) of experiment II (Table 4) (**A**, **C**) HLA-I positive cells; (**B**, **D**) CD45 positive cells.

). In blood, 0.9 to 6.6% human CD45 positive cells were found confirming the PCR results obtained several weeks before. In one example of experiment II, after 7 weeks no human DNA in the blood was detected. Testing the same mice after 11 weeks, a positive signal was obtained. That suggests a relatively long time period being necessary before establishment of a human haematopoiesis, leading to a detectable number of cells in the peripheral blood of mice.

In experiment III engraftment of CD34 cells completely failed probably due to unsufficient cell numbers. This led to completely negative results both in PCR, in FACS and IH.

The administration of 8×10^6^ CD34 cells gained from peripheral blood of patients undergoing mobilising chemotherapy (experiment IV) also revealed human-specific signals in PCR which were confirmed by FACS and IH analysis of blood and bone marrow cells. On contrary, a lower cell number of 3×10^6^, only led to a positive PCR result but failed to show the presence of human cells by FACS and IH. This emphasises the expected higher sensitivity of the PCR method compared to the other two techniques. All together, it can be concluded that the detection of human DNA in the peripheral blood of mice xenotransplanted with haematopoietic cells can be taken in the vast majority of cases as an indicator for a positive engraftment.

## DISCUSSION

After xenotransplantation of human cells into mice, it is frequently necessary to detect those cells as early as possible and at a relatively low level.

This problem for instance arises when metastasising models of human solid tumours are used (Giavazzi *et al*, 1986; Fodstad *et al*, 1988; Morikawa *et al*, 1988; Shoemaker *et al*, 1991) and their growth and dissemination potential has to be evaluated for an estimation of efficacy of therapeutic approaches.

Even more complicated is the situation when haematological malignancies as models in immunodeficient mice are used (Uckun, 1996). Here, frequently an early diagnosis of only few disseminated cells is warranted to start therapeutic interventions in a clinically-related manner as early as possible and before the disease becomes manifested and incurable.

Modern experimental gene- and immunotherapeutic approaches against cancer occasionally use transfected or modified haematological cells. In those pre-clinical studies it is inavoidable to characterise the survival and distribution potential of these cells (Licht *et al*, 1998).

And last but not least, several projects deal with the engraftment potential of blood stem cells for the enabling of high-dose-chemotherapy protocols. Questions of interest in this area are the potential of *ex vivo*-expansion protocols of CD34 positive cells (Möbest *et al*, 1999) or the role of stroma and endothelial cells for the engraftment of stem cells (Goan *et al*, 2000).

For that purpose, the necessity arose to develop an analytical method which is sensitive, specific and universally applicable to detect human cells in immunodeficient mice.

Shoemaker *et al* (1992) described the use of repetitive human Alu DNA sequences in a dotblot assay which allowed the quantitative detection of metastases of a human melanoma in several tissues of nude mice.

We decided to develop a human specific PCR method targeting highly repetitive sequences of the α-satellite DNA of the centromer region of human chromosome 17. A 850 bp fragment was amplified with primers which were varied according to Warburton *et al* (1991). A BLAST-search of this human sequence fragment in the EMBL rodent DNA database as well as in the ENSEMBLE whole mouse genome browser (EBI, Hinxton, Cambridge, UK) revealed no significant similarity with any mouse DNA sequence. Thus, these sequences seem to be completely absent in the mouse genome and the method shows high specificity for human tissues with a complete lack of positive signals for murine tissues. As shown by logarithmic dilution series, we were able to detect one human cell in 10^6^ murine cells. However, with 35 cycles necessary to reach the high sensitivity the method allowed only a rough semi-quantitative estimation. Therefore, we developed in parallel a quantitative real-time PCR approach based on TaqMan methodology, targeting a smaller fragment of the same genome localisation. The resulting standardised TaqMan assay provides the possibility for a quantification of human cells in the mouse from 100% up to 0.0001% (one human cell in 10^6^ mouse cells).

The method described herein was used for the detection of micrometastases of a breast carcinoma model. It proved its high sensitivity by detecting human DNA in lung and liver of NOD/SCID mice after orthotopic transplantation of only 10 cells and at an early time point when no primary tumour was palpable. The lack of positive signal in the blood of mice even at injection of up to 10^6^ cancer cells suggests the predominance of a lymphogenic route of dissemination leading to probably only few or shortly surviving cells in the blood circulation.

On the contrary, the manifestation of an ALL in SCID mice demanded at least 10^6^ cells and took several months. But even in this case, the PCR method allowed an about 50-days earlier detection of human cells in the blood of engrafted mice. That fact makes the xenotransplantation model suitable for the study of minimal residual disease and for the early commencement of therapeutic interventions.

Similar to others (Martino *et al*, 1993) we also stated that the survival of human mature lymphocytes gained from peripheral blood is rather limited, needing more than 10^7^ cells to be transplanted and requiring a conditioning of mice by sublethal total body irradiation. Those conditions have to be known for preclinical studies using immunotherapeutic agents (vaccines, antibodies).

Oppositely, haematopoietic stem cells isolated from cord blood or from cytapheresates of patients after mobilising chemotherapy under our conditions successfully engrafted in the majority of cases. Here, a good correlation of PCR, FACS and IH results could be shown.

In the past, imaging techniques have been developed for detection and quantification of tumours based on genetically engineered tumour cells expressing reporters such as beta galactosidase, green fluorescent protein or the newly emerging technology based on luciferase reporters (Rice *et al*, 2001). These methods have the relative disadvantage that they require transformation of tumour cells with an expression vector to enable detection and in the case of luciferase, infusion of the luciferin substrate intravenously. Such genetic manipulation could potentially alter phenotypes of relevance to experimental work. However, the imaging approaches are relevant to the current report in that both techniques could profitably be used in concert. For example, experiments using luciferase or GFP methods could be confirmed and generalised using our universally applicable PCR approach.

In summary, the PCR method described herein allows a specific and sensitive detection of human cells in xenotransplantation systems. The method is relatively simple, well-standardisable, non-radioactive, and except for the technical device relatively inexpensive. However, because of its high sensitivity, the method requires a very clean laboratory work to avoid any contamination with human genomic DNA. When performed as a real-time PCR using TaqMan methodology, it allows a reliable quantification of human cells in mouse organs over a wide dilution range. It can be used as an universally applicable surrogate marker for the quantitative evaluation of engraftment, proliferation and dissemination of malignant or normal cells in immunodeficient hosts.
